# Primary tubal choriocarcinoma with extrauterine implantation masquerading as ectopic pregnancy: A case report and literature review

**DOI:** 10.1097/MD.0000000000051163

**Published:** 2026-10-23

**Authors:** Ping Li, Yu Zhang, Jing Zhao, Xiaohui Zhao, Yiqi Jiang, Xia Yang, Peijuan Wang, Jie Fang

**Affiliations:** a Department of Gynecology, Affiliated Hospital of Jiangsu University, Zhenjiang, Jiangsu, China; b Department of Pathology, Affiliated Hospital of Jiangsu University, Zhenjiang, Jiangsu, China; c Department of Obstetrics and Gynecology, Affiliated Hospital of Integrated Traditional Chinese and Western Medicine, Nanjing University of Chinese Medicine, Nanjing, Jiangsu, China.

**Keywords:** chemotherapy, choriocarcinoma, gestational trophoblastic neoplasia, tubal ectopic pregnancy, β-hCG

## Abstract

**Rationale:**

Gestational trophoblastic neoplasia, particularly choriocarcinoma, is a rare but aggressive malignancy arising from trophoblastic tissue. Primary tubal choriocarcinoma is exceptionally uncommon and often presents a diagnostic challenge owing to its similarity to ectopic pregnancy.

**Patient concerns:**

A 29-year-old woman presented with irregular menses. Clinical suspicion of ectopic pregnancy was raised due to extremely high β-human chorionic gonadotropin levels and suggestive ultrasonographic findings.

**Diagnoses:**

Laparoscopic surgical exploration revealed a hemorrhagic mass involving the right fallopian tube and the pelvic sidewall. Histopathological examination confirmed a diagnosis of primary tubal choriocarcinoma with pelvic peritoneal implantation. The patient was classified as International Federation of Gynecology and Obstetrics stage III.

**Interventions:**

Following diagnosis, the patient underwent multiple cycles of combination chemotherapy, specifically 5-fluorouracil + actinomycin D.

**Outcomes:**

Serial β-human chorionic gonadotropin monitoring demonstrated an excellent response to treatment, with normalization within 2 months. The patient remained in sustained remission throughout the follow-up period.

**Lessons:**

This case highlights the diagnostic challenge of differentiating gestational choriocarcinoma from ectopic pregnancy, especially in the context of primary tubal involvement, and underscores the critical role of histopathological examination in establishing a definitive diagnosis.

## 1. Introduction

Gestational choriocarcinoma (GC) is a malignant form of gestational trophoblastic neoplasia (GTN) characterized by abnormal proliferation of trophoblastic cells.^[1]^ While most commonly arising from molar pregnancy, it can also follow term delivery, abortion, or ectopic pregnancy.^[2,3]^ Primary tubal choriocarcinoma is exceedingly rare, accounting for <1% of all GTN cases.^[4]^ Its clinical and radiological presentation often mimics that of ruptured ectopic pregnancy, posing diagnostic and therapeutic challenges. Delayed diagnosis may result in life-threatening hemorrhage and metastasis. Here,we report a case of primary tubal choriocarcinoma treated with pelvic implantation. Initially,ectopic pregnancy was suspected to raise awareness regarding this rare condition.

## 2. Case presentation

A 29-year-old woman, gravida 1, para 1, with a history of irregular menstrual cycles (7/30–60 days) presented in June 2024 with 74 days of amenorrhea. A right adnexal mass was discovered one day prior to admission. Her last menstrual cycle was on March 18, 2024. A self-administered urine pregnancy test was negative one month after amenorrhea but exhibited a weakly positive result on June 2, 2024, indicating the possibility of early pregnancy or the need for additional testing to confirm the status. External ultrasound indicated a normal endometrial thickness of 7 mm, which may correspond to mid-cycle preparation for ovulation without evidence of an intrauterine gestational sac. A 63 × 53 mm hyperechoic mass was noted adjacent to the right ovary, raising the suspicion of ectopic pregnancy. She denied experiencing abdominal pain, vaginal bleeding, or any other symptom.

Physical examination results were unremarkable,except for a thickened right adnexa without tenderness. Transvaginal ultrasound examination at our hospital revealed a 72 × 61 × 50 mm heterogeneous, hypervascular mass in the right adnexa, along with a small amount of pelvic fluid (Fig. 1). Given its characteristics, this mass could be indicative of a complex adnexal lesion,which requires further evaluation to determine its nature. The serum β-human chorionic gonadotropin (β-hCG) level was significantly elevated at 293,499 mIU/mL, which is considerably higher than the normal range for early pregnancy and may suggest multiple pregnancies, molar pregnancies, or other pathological conditions.

**Figure 1.**
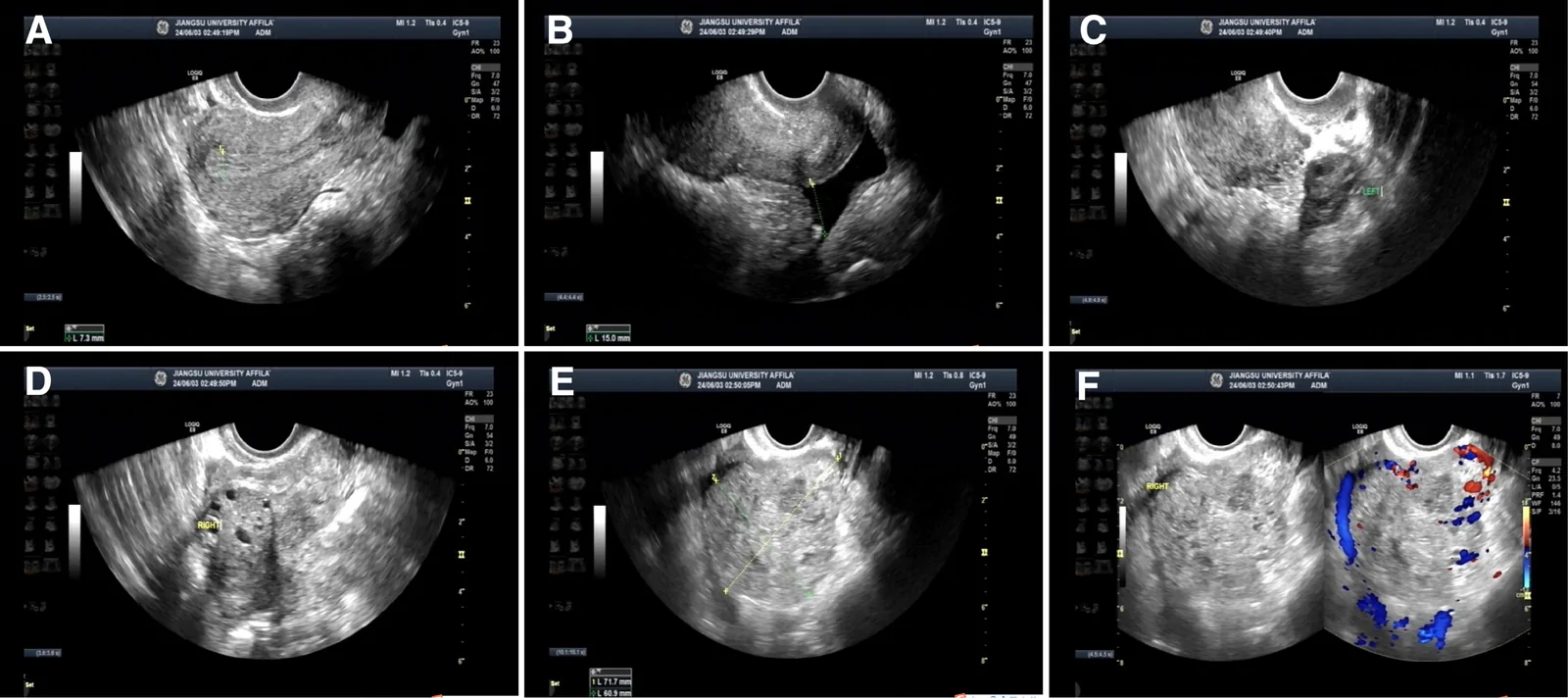
Transvaginal ultrasound. (A) Transvaginal ultrasound shows an endometrial thickness of 7 mm; no gestational sac is visualized within the uterine cavity. (B) A 16-mm depth of anechoic fluid is present in the posterior uterine pouch (arrow). (C) Sonographic appearance of the left adnexa. (D–F) Transvaginal gray-scale and color Doppler images of the right adnexa revealing a slightly hyperechoic mass measuring 71.7 × 60.9 × 50 mm with heterogeneous internal echoes. Color Doppler (CDFI) demonstrates abundant blood flow both within and along the periphery of the lesion (arrows).

Exploratory laparoscopy was performed on June 3, 2024. The uterus was slightly enlarged. The right fallopian tube appeared normal;however, active bleeding was observed at the fimbriated end of the tube. A large (7 × 6 × 6 cm), irregular, bluish-purple soft tissue mass was found to be adherent to the broad ligament and pelvic sidewall, enveloping the right ovary. Approximately 100 mL of hemoperitoneum was observed. The mass was densely adherent to the pelvic sidewall with extensive active bleeding. A right salpingectomy and extensive resection of the pelvic mass were performed. The surgery lasted 155 minutes, with an estimated blood loss of 400 mL (Fig. 2).

**Figure 2.**
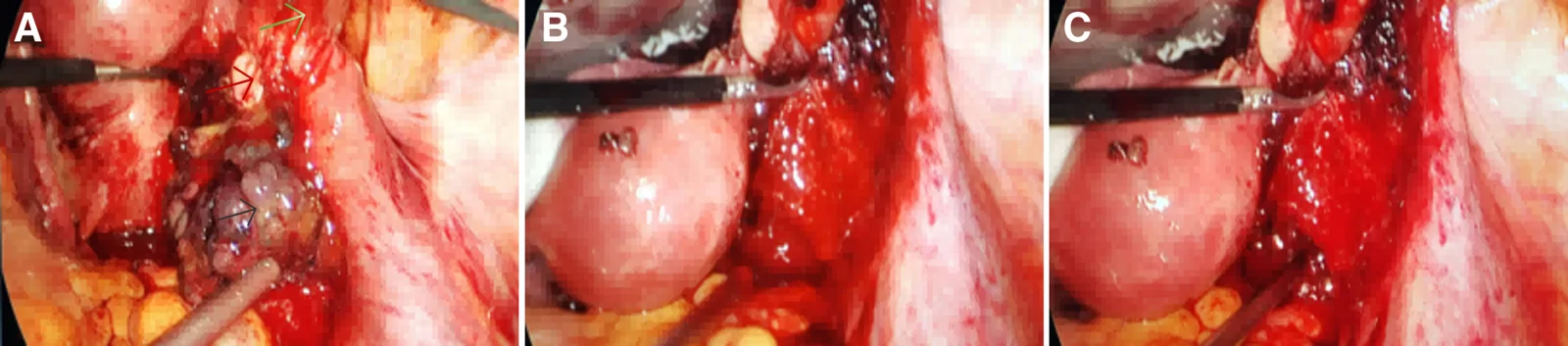
Laparoscopic exploration. The black arrow points to the peritoneal lesion; the green arrow indicates the right ovary; the red arrow marks the right fallopian tube.

Histopathological Findings:Microscopic examination revealed scattered, multifocal deposits of tumor cells on the serosal surface of the fallopian tube and pelvic peritoneum, which is consistent with the potential for primary tumors to arise from the peritoneum, as discussed in the literature. The tumor was composed of a trimorphic population of malignant trophoblastic cells: intermediate trophoblasts, cytotrophoblasts, and syncytiotrophoblasts, which are often arranged in a classic biphasic pattern with syncytiotrophoblasts surrounding the cytotrophoblasts. Chorionic villi, intervening stroma, and intrinsic vascular structures were absent.

The tumor cells were arranged in solid sheets and exhibited marked nuclear atypia characterized by hyperchromatic nuclei, prominent nucleoli, and frequent mitotic figures. Extensive areas of hemorrhage and necrosis were present adjacent to the tumor. Definitive lymphovascular invasion was not observed in any of the examined sections.

All the tumor cells showed diffuse expression of cytokeratin AE1/AE3. The Ki-67 proliferation index was approximately 90%, indicating a high level of tumor cell proliferation, which is typically associated with more aggressive tumor behavior and poorer outcomes. Syncytiotrophoblasts showed diffuse and strong hCG expression, whereas intermediate trophoblasts and cytotrophoblasts showed weak expression. Intermediate trophoblasts were characteristically positive for mucin-4. The tumor cells were negative for placental alkaline phosphatase (Fig. 3). The negative p57 staining, coupled with the histopathological findings, confirmed the gestational origin of the choriocarcinoma, consistent with previous reports_._^[5]^

**Figure 3.**
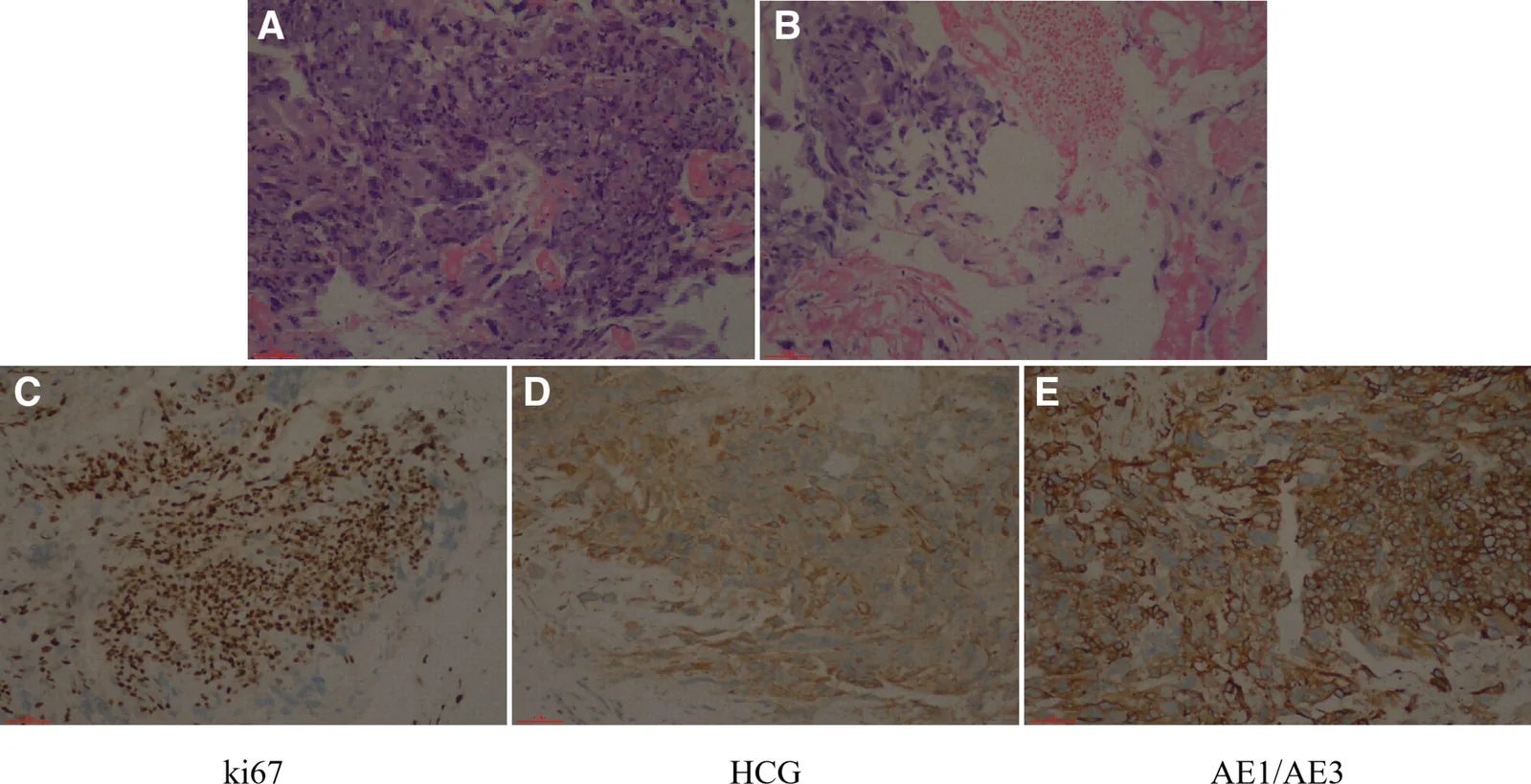
Histopathological and immunohistochemical characteristics of choriocarcinoma. (A) Tumor: Microscopic examination revealed a classic biphasic pattern of choriocarcinoma, composed of abundant mononuclear trophoblasts (cytotrophoblasts and intermediate trophoblasts) intermixed with multinucleated syncytiotrophoblasts. (B) Tumor with hemorrhage and necrosis: Extensive areas of hemorrhage and necrosis are present adjacent to sheets of tumor cells. (C) AE1/AE3: Diffuse and strong cytoplasmic immunoreactivity for the pan-cytokeratin marker AE1/AE3 was observed in all tumor cells, confirming the epithelial origin of the neoplasm and supporting the diagnosis of carcinoma. (D) hCG: syncytiotrophoblasts show strong and diffuse cytoplasmic expression of hCG. A subset of intermediate trophoblasts showed weak positivity. (E) Ki-67: The tumor exhibited a high proliferative index, with approximately 90% of the neoplastic cells exhibiting intense nuclear staining for Ki-67, indicating highly aggressive tumor behavior and correlating with a poor prognostic profile.

Postoperative staging involved chest computed tomography,which revealed small pulmonary nodules. While these nodules were not indicative of metastasis, their significance requires further evaluation, considering factors such as size, shape, and doubling time, as well as the patient’s clinical history and risk factors. The final diagnosis was stage IV choriocarcinoma, characterized by widespread metastasis to distant organs, such as the brain, liver, and kidneys, as per the International Federation of Gynecology and Obstetrics (FIGO) 2000 staging system,^[6]^ which developed secondary to tubal pregnancy.The patient was referred to the oncology department, where staging was revised to Clinical Staging FIGO^[7]^: FIGO Stage III (extension to pelvic structures beyond the primary site). World Health Organization Prognostic Score^[8,9]^: 7 (high-risk) based on age (0), antecedent pregnancy (ectopic, 0), interval (0), β-hCG level (4), largest tumor size (2), site of metastasis (0), number of metastases (1), and previous chemotherapy (0). She received her first cycle of 5-fluorouracil + actinomycin D (FA) chemotherapy (actinomycin-D 0.5 mg d1-5 and 5-FU 5g continuous intravenous infusion over 120 hours) on June 13, 2024, with prophylactic pegylated granulocyte colony-stimulating factor support. The patient subsequently received multiple additional cycles of FA chemotherapy on July 10, August 6, August 31, and September 21, 2024. Serial β-hCG levels demonstrated a rapid and excellent response (Table 1).

**Table 1:** Serial β-hCG levels demonstrated a rapid and excellent response.

Monitoring of the serum β hCG levels during perioperative stage		
DATE	β hCG (mIU/mL)	NOTE
Operation day		
2024/6/3	293,499	No exact value
1st day after operation		
2024/6/4	183,127	No exact value
5th day after operation		
2024/6/8	47,408	
The first chemotherapy session		
2024/6/12	2192	
2024/6/27	640.3	
2024/7/4	128.2	
The second chemotherapy session		
2024/7/9	51.93	
2024/7/18	16.96	
2024/7/25	8.6	
The third chemotherapy session		
2024/8/5	3.92	Normal
2024/8/14	2.07	
The fourth chemotherapy session		
2024/8/30	1.1	
The fifth chemotherapy session		
2024/9/20	0.82	
2024/9/30	0.54	
2024/10/14	0.48	
2024/11/3	0.14	
2024/11/17	<0.100	
2024/12/1	<0.100	
2024/12/15	<0.100	
2024/12/29	<0.100	
2025/1/12	<0.100	
2025/1/26	<0.100	
2025/2/9	<0.100	
2025/3/2	<0.100	
2025/3/30	<0.100	
2025/4/27	<0.100	
2025/5/25	<0.100	
2025/6/29	<0.100	
2025/7/27	<0.100	
2025/8/24	<0.100	
2025/10/19	<0.100	

β-hCG = β-human chorionic gonadotropin.

The β-hCG levels remained within the normal range (<0.100 mIU/mL) during subsequent chemotherapy and throughout the follow-up period until the latest test in October 2025, confirming sustained complete remission. A transient, minimal elevation of 0.95 mIU/mL was noted in March 2025 but promptly normalized in subsequent tests. The patient also had elevated alpha-fetoprotein (AFP) levels during follow-up, which were being monitored.

## 3. Discussion

Primary fallopian tube choriocarcinoma is an exceptionally rare and diagnostically challenging form of GTN. In the present case, the initial presentation of amenorrhea, markedly elevated β-hCG, and an adnexal mass closely mimicked a common ectopic pregnancy, making preoperative differentiation extremely difficult. A review of the 10 previously reported cases summarized in Table 2 further indicates that this entity most often occurs in women of reproductive age and typically presents with nonspecific symptoms, including amenorrhea, abnormal vaginal bleeding, abdominal pain, and elevated serum β-hCG levels. Because these features substantially overlap with those of ectopic pregnancy, preoperative misdiagnosis is common. Although findings such as extremely elevated β-hCG levels (typically exceeding 100,000 mIU/mL), a large hypervascular adnexal mass on Doppler ultrasonography, and evidence of metastatic disease may raise suspicion for malignancy, definitive diagnosis still relies on histopathological examination demonstrating biphasic cytotrophoblasts and syncytiotrophoblasts without chorionic villi, supported by immunohistochemical findings such as β-hCG positivity, anti-his tag antibody (clone 3D5) positivity, and p57 negativity.Surgical resection followed by postoperative chemotherapy remains the mainstay of treatment, and the prognosis is generally favorable when early diagnosis and timely multimodal therapy are achieved (Table 2).^[10–19]^

**Table 2:** Clinical characteristics, treatment strategies, and outcomes of previously reported cases of primary tubal choriocarcinoma.

First author	Age	Clinical presentation	β-hCG (mIU/mL)	Tumor size (cm)	Surgical treatment	Chemotherapy regimen	Outcome
Bhanji et al^[10]^	28	Amenorrhea for 4 mo; ultrasonography revealed a solid mass in the left adnexa	29,116	10 × 10 × 9	Resection of the adnexal mass	MTX	No metastases were observed during follow-up.
Mann et al^[11]^	37	Abdominal pain and spotting, progressing to heavy menstrual bleeding 1 mo later	>1,000,000	8 × 7 × 12	Total abdominal hysterectomy and bilateral salpingo-oophorectomy	None	No postoperative follow-up data were available.
Najib et al^[12]^	34	Vaginal bleeding for 1 wk and right lower abdominal pain	14,000	2 × 1.5	Right salpingectomy	EMA-CO	No recurrence was observed during 1-yr follow-up.
Wan et al^[13]^	54	Irregular vaginal bleeding for 2 mo after 3 mo of amenorrhea	291,116	Imaging revealed a cystic-solid adnexal mass	Hysterectomy and bilateral salpingo-oophorectomy	5-Fu + KS	No recurrence was observed during a 14-mo follow-up.
Gálvez et al^[14]^	33	Severe pain in the right iliac fossa accompanied by mild genital tract bleeding	142.1	3.73	Right salpingectomy	MTX + Act-D + VCR	No recurrence was observed during a 2-yr follow-up.
Karaman et al^[15]^	31	Amenorrhea, left lower abdominal pain, and fatigue	29,251.4	4 × 3	Left salpingectomy	MTX	β-hCG levels gradually decreased to negative during follow-up.
Xu et al^[16]^	42	Amenorrhea for 37 d, vaginal bleeding for 1 wk, and severe lower abdominal pain for half a day	>100,000	6 × 5	Salpingectomy on the affected side	5-FU + Act-D + VCR	No recurrence was observed during 1-yr follow-up.
Lee et al^[17]^	31	A gravida at 32 + 3 wk of gestation presented with dyspnea and blood-tinged sputum	228,300	7 × 6 × 3	Cesarean section combined with tumor resection, left salpingectomy, and omentectomy	Two cycles of EMA-CO, then switched to EMA-CE because of neurotoxicity	At 16 mo after treatment, both the mother and infant remained alive and disease-free.
Dekel et al^[18]^	22	Lower abdominal pain for 2 wk; amenorrhea for 8 wk	95	11 × 8 × 5	Adnexectomy and bilateral theca-lutein cystectomy	Yes	Two years later, the patient delivered a healthy infant and remained alive and in good health at the 5-yr follow-up.
Dasari et al^[19]^	20	Amenorrhea for 5 mo, abdominal pain for 3 d, and vaginal bleeding for 1 d	None	10 × 12	Left adnexectomy and bilateral internal iliac artery ligation	EMA-CO	Uneventful recovery.

5-Fu = 5-fluorouracil, Act-D = actinomycin D, EMA-CE = etoposide + methotrexate + actinomycin-D + cyclophosphamide + etoposide, EMA-CO = etoposide + methotrexate + actinomycin-D + cyclophosphamide + vincristine, KS = dactinomycin, MTX = methotrexate, VCR = vincristine, β-hCG = β-human chorionic gonadotropin.

The management of GTN predominantly relies on chemotherapy, which is effective even in advanced stages owing to the high chemosensitivity of the tumor.^[20]^ Staging discrepancies (revised FIGO Stage III) highlight the need for comprehensive evaluation, including imaging and histopathology, according to FIGO guidelines. The lung micronodules raised metastatic concerns but were likely benign, as they did not progress. Maximal surgical debulking, as demonstrated in the treatment of glioblastoma multiforme and non-small cell lung cancer, is crucial for hemorrhage control, cytoreduction, and tumor burden reduction before chemotherapy, thereby enhancing the efficacy of subsequent treatments.^[7,8]^ The FA regimen, an etoposide-free alternative to EMA-CO (etoposide, methotrexate, actinomycin D, cyclophosphamide, and vincristine), was selected, possibly because of patient factors or institutional protocols, as evidenced by a study comparing 5-fluorouracil + actinomycin D + etoposide + vincristine and EMA/CO regimens for GTN, which achieved an excellent response, with >99% β-hCG decline within months.^[21]^ The FA regimen (5-FU + actinomycin-D) is a first-line treatment option for GTN, with a reported complete response rate of 93.18% in one study and is particularly effective in medium- or high-risk cases.

The swift normalization of our patient’s β-hCG levels post-surgery and following the initial 2 cycles of chemotherapy indicated a highly favorable treatment response. Sustained normalization of β-hCG levels for more than a year post-treatment is a strong indicator of cure, as evidenced by clinical studies involving 152 patients with persistent tumors post-treatment, where a significant portion experienced no change or regression of metastatic lesions.

The transient, minimal increase in β-hCG (0.95 mIU/mL) on March 2025 highlights the importance of vigilant follow-up. Such “phantom hCG” or small fluctuations can occur but do not necessarily indicate relapse if they promptly normalize without a rising trend.^[22]^ AFP elevation is not a typical feature of pure choriocarcinoma and is more commonly associated with yolk sac tumor and embryonal carcinoma. However, AFP may be elevated in choriocarcinoma under certain circumstances, such as mixed germ cell tumors containing AFP-producing components, extragonadal tumors with mixed histology, or GC, in which disruption of the placental barrier may permit fetal-derived AFP to enter the maternal circulation. In the present case, additional evaluation, including liver function tests, hepatic ultrasonography, and abdominal computed tomography, revealed no evidence of liver disease or a second primary tumor. Therefore, the AFP elevation in this patient remains unexplained but was unlikely to be related to hepatic pathology or an alternative AFP-producing malignancy.

## 4. Conclusion

This case underscores that choriocarcinoma should be considered in the differential diagnosis of women of reproductive age presenting with a complex adnexal mass and very high β-hCG levels even when ectopic pregnancy is suspected. A multidisciplinary approach involving gynecology, oncology, and pathology is crucial. Timely surgery for the diagnosis and control of hemorrhage, followed by appropriate chemotherapy based on risk stratification, can significantly improve survival rates and quality of life, as evidenced by studies indicating that even in advanced-stage diseases such as gastric cancer, the success rate of surgery can be increased to 80 to 90% with neoadjuvant chemotherapy. Long-term β-hCG surveillance is essential to confirm remission.

## Acknowledgments

The authors would like to thank the Department of Gynecology and Oncology at Affiliated Hospital of Jiangsu University for their clinical support and assistance in the diagnosis and management of this patient. We are also grateful to the patient and her family for providing informed consent for the publication of this case report.

## Author contributions

**Conceptualization:** Ping Li, Peijuan Wang.

**Data curation:** Ping Li, Xiaohui Zhao, Jie Fang.

**Investigation:** Ping Li, Yu Zhang, Jing Zhao, Xiaohui Zhao, Yiqi Jiang, Xia Yang, Peijuan Wang.

**Visualization:** Ping Li, Xiaohui Zhao, Xia Yang, Jie Fang.

**Writing – original draft:** Ping Li, Peijuan Wang, Jie Fang.

**Writing – review & editing:** Ping Li, Yu Zhang, Jing Zhao, Xiaohui Zhao, Yiqi Jiang, Xia Yang, Peijuan Wang, Jie Fang.
